# Increased root hair density by loss of *WRKY6* in *Arabidopsis thaliana*

**DOI:** 10.7717/peerj.2891

**Published:** 2017-01-24

**Authors:** Markus G. Stetter, Martin Benz, Uwe Ludewig

**Affiliations:** 1 Institute of Crop Science, Nutritional Crop Physiology, University of Hohenheim, Stuttgart, Germany; 2 Institute of Plant Breeding, Seed Science and Population Genetics, University of Hohenheim, Stuttgart, Germany

**Keywords:** *Arabidopsis*, Root hair, Ecotypes, Genome wide association, Mixed model, Nutrition, Phosphorus, Inorganic phosphate, Cortex, Epidermis

## Abstract

Root hairs are unicellular elongations of certain rhizodermal cells that improve the uptake of sparingly soluble and immobile soil nutrients. Among different *Arabidopsis thaliana* genotypes, root hair density, length and the local acclimation to low inorganic phosphate (P_i_) differs considerably, when analyzed on split agar plates. Here, genome-wide association fine mapping identified significant single nucleotide polymorphisms associated with the increased root hair density in the absence of local phosphate on chromosome 1. A* loss-of-function*mutant of the candidate transcription factor gene *WRKY6*, which is involved in the acclimation of plants to low phosphorus, had increased root hair density. This is partially explained by a reduced cortical cell diameter in *wrky6-3*, reducing the rhizodermal cell numbers adjacent to the cortical cells. As a consequence, rhizodermal cells in positions that are in contact with two cortical cells are found more often, leading to higher hair density. Distinct cortical cell diameters and epidermal cell lengths distinguish other *Arabidopsis* accessions with distinct root hair density and −P_i_ response from diploid Col-0, while tetraploid Col-0 had generally larger root cell sizes, which explain longer hairs. A distinct radial root morphology within *Arabidopsis* accessions and *wrky6-3*explains some, but not all, differences in the root hair acclimation to –P_i_.

## Introduction

The essential macronutrient phosphorus is mainly taken up by plant roots as inorganic ortho-phosphate (P_i_). Phosphate is sparingly soluble in soil and forms plant-unavailable precipitations with iron and aluminum at low acidic pH. The plant available P_i_ is also low at alkaline pH, where P_i_ is bound to calcium. Phosphate is absorbed and bound to soil particle surfaces, which drastically reduces the P_i_ soil mobility, so that mass flow contributes very little to P_i_ nutrition (Marschner, 2011). As a consequence, plant roots not only use sophisticated mechanisms to increase the solubility of soil phosphate, but also form fine roots in soil areas close to the surface, where the highest P_i_ concentrations are found (Vance, Uhde-Stone & Allan, 2003). As the P_i_ diffusion in soil is very low, the root surface area is further increased at low P_i_ by promoting root hair growth and density (Müller & Schmidt, 2004). Efficient P_i_ usage by crops is of crucial importance as global high quality rock phosphate reserves are estimated to be depleted within the next 100-400 years (Cordell, Drangert & White, 2009; Van Kauwenbergh, 2010), imposing challenges for future crop production.

Root hairs are formed just basal to the root expansion zone behind the tip to increase the water and nutrient uptake. Various, more or less regular patterns of hair arrangements, are found in different species, with the model plant *Arabidopsis thaliana* having a simple, regular pattern ideally suited for genetic dissection of the underlying genetics (Datta et al., 2011). Root hairs provide a competitive advantage to *Arabidopsis thaliana* (Bates & Lynch, 2001) and barley (Gahoonia & Nielsen, 2003) under low P_i_ availability, by increasing the acquisition of P_i_. When grown in artificial rockwool substrate, *Arabidopsis* mutants that lack root hairs had decreased water absorption and lowered drought tolerance (Tanaka et al., 2014). The uptake of several minerals was decreased by the lack of root hairs and the secretion of acid phosphatase and organic acids was reduced. Phosphatase and organic anion exudation are physiological rhizosphere strategies to solubilize unavailable, organically bound P_i_ and acquire P_i_ by anion ligand exchange, respectively (Tanaka et al., 2014).

In *Arabidopsis thaliana*, epidermal root hair or non-root hair cells develop after asymmetric divisions from epidermal precursor cells. The root hair length and density is regulated by several other nutrients besides phosphorus, such as iron and manganese (Ma et al., 2001). Furthermore, nitrate stimulates hair density, while ammonium has less effect, but nutritional effects are genotype-dependent (Vatter et al., 2015). The plant hormones auxin, ethylene and strigolactones (Kapulnik et al., 2011; Masucci & Schiefelbein, 1994; Pitts, Cernac & Estelle, 1998; Rahman et al., 2002) control both root hair length and density, probably via their complex effects on general root growth.

Phosphorus deficiency leads to local and systemic transcriptional responses in root cells (Lan, Li & Schmidt, 2012; Thibaud et al., 2010). *PHO1* encodes a major component of the root to shoot P_i_ translocation machinery and is a central component of the P_i_ homeostasis network (Rouached et al., 2011; Wege et al., 2015). *PHO1* expression is regulated by the transcription factor *WRKY6* (Chen et al., 2009).

In the primary root, local P_i_ deficiency reduces meristem activity, which in turn suppresses primary root growth, leading to shorter rhizodermal cells. This increases the longitudinal frequency (density) of root hairs. In radial root sections of *Arabidopsis,* root hairs are mostly found in hair H-positions, in which the epidermal cells are in contact with two cortical cells. A positional peptide signal exported from the cortical cells is involved to determine the fate of hair cells (Savage et al., 2013). Rhizodermal cells that are in so called non-hair N-positions have only contact with a single cortex cell and do not develop into hair cells. However, ectopic root hairs that develop in N-positions may also contribute to the higher density of root hairs under P_i_ starvation in *Arabidopsis* (Müller & Schmidt, 2004; Savage et al., 2013).

By contrast, after initiation, the elongation time of tip-growing root hairs determines their final length. The length is critically dependent on the activity of the transcription factor ROOT HAIR DEFECTIVE 6-LIKE 4 (RSL4), which is necessary and sufficient for root hair growth (Datta, Prescott & Dolan, 2015). The elongation time and the growth rates are promoted under P_i_ deficiency, leading to longer root hairs in the *Arabidopsis* accession Col-0 (Bates & Lynch, 1996). Root hair density and length varies strongly among *Arabidopsis* accessions and surprisingly, individual accessions with strongly reduced hair numbers in −*P*_*i*_ were encountered, at least when grown on split agar plates (Stetter, Schmid & Ludewig, 2015). Furthermore, isogenic tetraploids had increased hair numbers, a phenomenon that might be caused by larger root cell sizes, which are commonly found in polyploids, or alternative radial root architecture.

Using a recently developed setup for *Arabidopsis* root growth from a fully supplied medium (to ensure sufficient initial nutrient supply) into a medium without P_i_, we hypothesized that the different root hair densities and responses to the lack of P_i_ were caused by different radial root morphologies. Re-analysis of root hair data from a subset of accessions (Stetter, Schmid & Ludewig, 2015) identified a novel chromosomal candidate region close to *WRKY6* for root hair density in −P_i_. The different hair densities and −P_i_ responses in contrasting *Arabidopsis* accessions and in a *wrky6* mutant were partially explained by distinct root morphologies.

## Material and Methods

### Plant material

*Arabidopsis thaliana* L. genotypes were chosen on basis of previous results. The ecotypes Ga-0 and Kondara were chosen because of their strong root hair length and density adaptation to low P in a pervious study (Stetter, Schmid & Ludewig, 2015). * wrky6* mutants were obtained from T. Fujiwara (Kasajima et al., 2010) and tetraploid Col-0 (4×) seeds were obtained from D. Chao (Chao et al., 2013) to study the influence of WRKY6 and polyploidy on root hair traits. Col-0 was included as reference and background of *wkry6* mutants.

### Split plates and growth conditions

Vertically placed split squared Petri dishes (120 mm × 120 mm × 17 mm, Greiner bio-one, Kremmünster, Austria) with a top compartment containing all nutrients, and a lower test compartment with or without P_i_ (KH_2_PO_4_ substituted by KCl) were used. The growth medium was composed of 5 mM KNO_3_, 2 mM MgSO_4_, 2 mM Ca(NO_3_)_2_, 70 µM HBO_3_, 14 µM MnCl_2_, 1 µM ZnSO_4_, 0.5 µM CuSO_4_, 10 µM NaCl, 0.2 µM Na_2_MoO_4_, 40 µM FeEDTA, 4.7 mM MES and 1 mM KH_2_PO_4_, 43 mM sucrose, pH was adjusted to 5.7 with 1 M KOH, solidified with 1.2% phytoagar.

Seeds were derived from plants grown in nutrient-rich garden soil (Einheitserde EET Einheitserde- und Humuswerke, Sinntal-Jossa, Germany), and surface sterilized with 70% ethanol and 0.05% TritonX 100 for 15 min under continuous shaking and were washed twice with 70% ethanol for 5 min. After washing, the seeds were dried on filter paper under a clean bench. Eight to fifteen seeds were placed 1 cm above the split. Plates were stored for two days at 4°C in darkness, before being kept under 21°C and continuous light for 12 h. After light treatment, plates were kept in cold and darkness for another day before being transferred into the growth chamber. Plants were grown under constant light (180 µE) and 21°C.

### Image acquisition and analyses

For each accession replicated images of six to ten roots per treatment were taken with 20× magnification with a Zeiss AxioCam MRm (Carl Zeiss, Jena, Germany) monochrome camera under a Zeiss Stemi 2000-c binocular microscope (Carl Zeiss, Jena, Germany) to assess root hair length and density. Root hairs were measured and counted using ImageJ (http://imagej.nih.gov). To investigate the root cell length and diameter, roots were treated with a 1:250 diluted propidium iodide (2.5 mM) solution in water. Roots were stained for 210 s, before being washed. Images were acquired 1 cm above the root tip, where root hairs were fully developed, once the root was 3 cm long. Images were taken at 20× magnification with a Zeiss LSM 700 microscope (Carl Zeiss, Jena, Germany). 3D image stacks were performed to observe the cross sections. The interval between the stacks was adjusted between 1.5 and 2 µm. Epidermal cell length, root diameter, cortex cell diameter, epidermal cell diameter, and the number of epidermal cells adjacent to each cortex cell were analyzed using ImageJ (http://imagej.nih.gov). For measuring the epidermal cell length, a representative epidermal cell being in contact with two cortical cells was measured for each sample. Ectopic root hairs (in non-hair positions) were encountered very rarely, not sufficient to be included in statistical analysis. For measuring the cortex cell diameter, the means of three neighboring cortex cells were measured. The epidermal cell diameter was quantified from the epidermal cells adjacent to these three cortical cells.

### Statistical data analysis

A mixed linear model was used for data analysis, reflecting the experimental design. The following model was employed for the diversity study:

\begin{eqnarray*}Y=\mathrm{GEN}\times \mathrm{TRT}:\text{(REP/PLATE)/PLANT} \end{eqnarray*}

where, *Y* = Response variable (root hair density or root hair length), GEN = Fixed effect of the genotype, TRT = Fixed effect of the treatment, REP = Random effect of the replicate, PLATE = Random effect of the Petri dish, PLANT = Residual effect of the plant, *x* = main effects + interaction, / = nested relationship.

Data analysis was performed with the MIXED procedure in SAS/STAT of SAS^®^ 9.3. Variance homogenity was reviewed and outliers were identified and removed based on studentized residuals. Values with studentized residuals larger than 4 were removed from the dataset.

### GWA mapping

Associated SNPs with Bonferroni corrected *p*-values smaller than 0.0001 were analyzed for gene identification based on the TAIR10 gene annotation (www.arabidopsis.org). We used sequence data of 71 accessions to analyze the candidate region on chromosome 1 with higher resolution. A window of 20,000 bp up- and downstream of the associated SNP was extracted and the genomic data were imputed using MaCH before SNPs with allele frequencies <0.05 were dismissed. Association mapping was performed with MLMM (Segura et al., 2012), to apply association mapping on the genomic region around a SNP, which previously has been found in high association with root hair density adaptation to local P_i_ scarcity (Stetter, Schmid & Ludewig, 2015).

The experiment to evaluate the root hair length and density of *wrky6* was laid out as split- plot design with three replicates. For the root cell measurement, a split-plot design with two complete replicates was chosen. For each replicate one petri dish per treatment and accession was prepared and four plants per petri dish were sampled.

## Results

### Genome-wide association mapping of root hair density and response to absence of local P_i_

**Figure 1:**
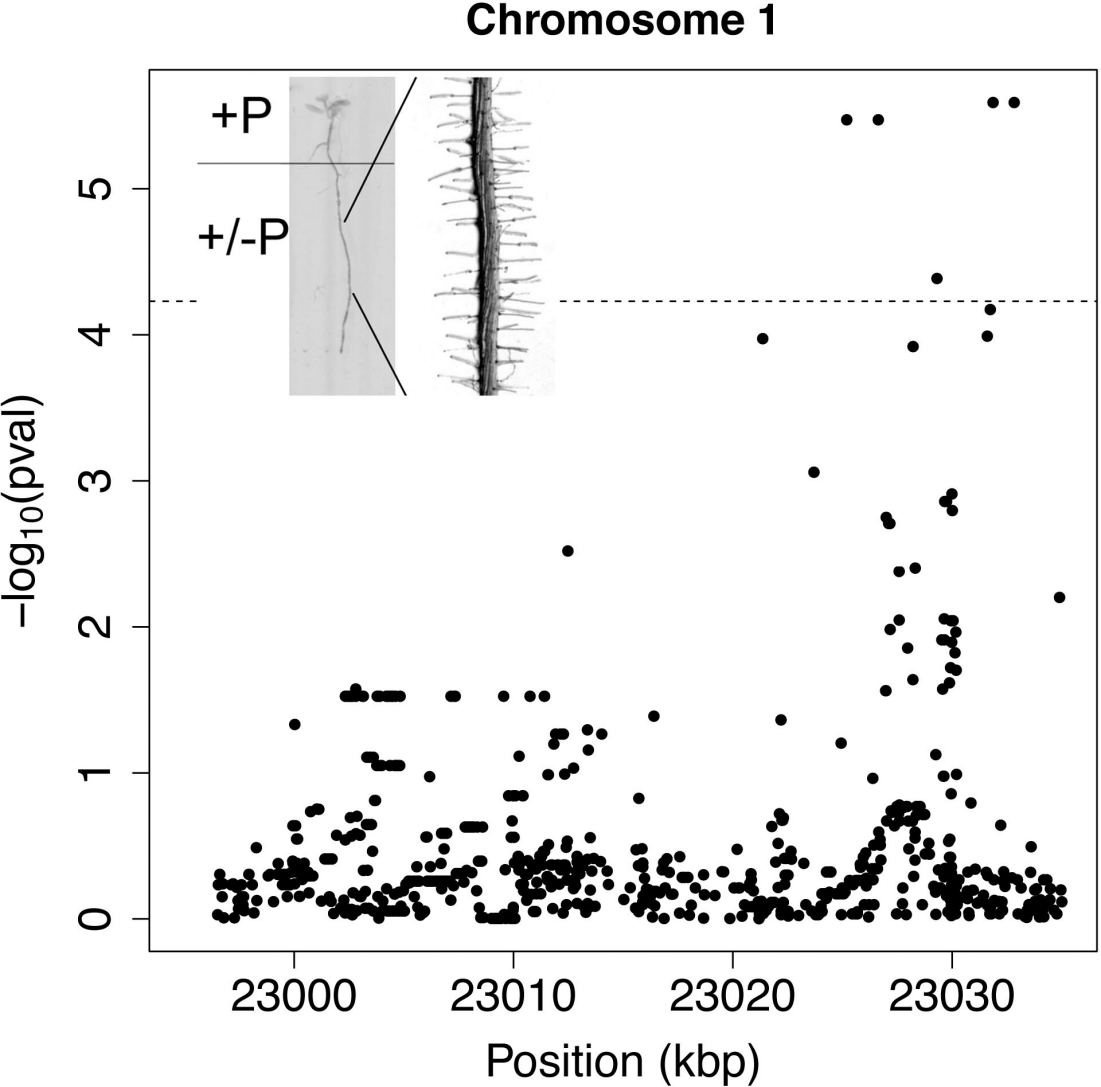
Manhattan-plot of fine mapping with whole genome data of 71 accessions to root hair density response. Window of genomic region 20 kb up- and down-stream of *WRKY6* on Chromosome 1. The horizontal line indicates significant Bonferroni corrected *p*-values at 5% significance level. Inset: Sketch of the experimental design of split plates with seeds positioned on the top +P_i_ compartment.

Root hairs of more than 160 *Arabidopsis* accessions were recently phenotyped (Fig. 1, inset) on vertical split agar plates (Stetter, Schmid & Ludewig, 2015). Genome wide association (GWA) mapping had identified candidate genes potentially involved in root hair density and surface acclimation to the absence of P_i_. However, mutant analysis with few candidate genes did not identify direct relation to P_i_ sensing. Rather, mutations in candidate genes typically increased root hair densities and imposed P_i_ insensitivity, suggesting that these genes repress root hair density under P_i_ availability, rather than promote hair density in the absence of P_i_ (Stetter, Schmid & Ludewig, 2015).

**Figure 2:**
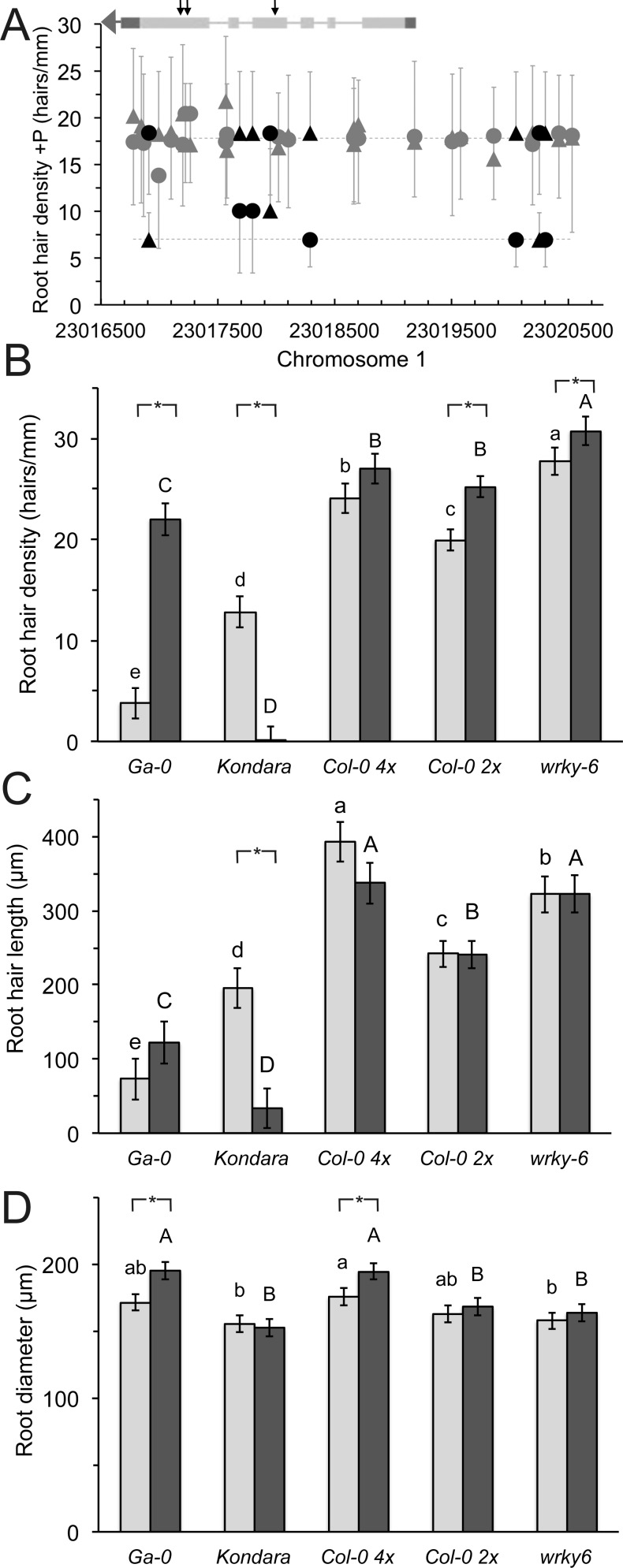
Root hair length, density and diameter of selected *Arabidopsis thaliana* accessions and a mutant in +*P*_*i*_ and −P_*i*_ conditions. (A) Mean hair density under control conditions associated with different alleles (squares or circles) in the *WRKY6* gene and promoter (at 1.3 kb the next gene is encoded). *WRKY6* is encoded in antisense orientation, untranslated regions and exon-intron structure are given in the inset, arrows indicate SNPs that lead to amino acid changes. Alleles with significant density differences are drawn in black. Error bars indicate standard deviation. Root hair (B) length, (C) density and (D) root diameter of Ga-0, Kondara, Col-0 2×, Col-0 4×, *wrky6-3*. Light gray bars: +P_i_, dark grey bars: −P_i_. Error bars indicate standard error. Capital letters indicate significant differences (*p* < 0.05) in −P_i_, lower case letters indicate significant differences in +P_i_. Significant differences between +P_i_, and −P_i_ are given as *.

While re-evaluating previous data, a non-significantly associated SNP in close proximity to *WRKY6* captured our attention. *WRKY6* encodes a transcription factor that physically interacts with the *PHO1* promoter and is involved in Pi homeostasis (Chen et al., 2009). While the SNPs associated with *WRKY6* did not have significant *p*-values in the original GWA mapping (Stetter, Schmid & Ludewig, 2015), fine mapping with 71 accessions for which whole genome sequences were available identified five highly significant SNPs 6 to 13 kb upstream to *WRKY6* (Fig. 1). Furthermore, the coding sequence of *WRKY6* was polymorphic at 11 positions among these 71 sequenced genotypes, of which three nucleotide exchanges lead to amino acid changes: 250V/G, 443S/G and 450V/A. These alleles were encountered at frequencies between 6–23% When calculated for individual alleles, the mean density and length of these alleles was similar, indicating that these polymorphisms are unlikely affecting *WRKY6* function, but the mean hair density was significantly different for a few low frequency alleles (4–6%) in the promoter, in introns or in exons. This was most obvious under control conditions (Fig. 2D) and resulted in different responsiveness of these alleles.

### Root hair length and density differences in *wrky6-3* and other *Arabidopsis* accessions

The root hair density, length, root diameter and the response to scarce local P_i_ were subsequently analyzed with a *wrky6-3* mutant, to address a potential further function of this gene in P_i_-related root hair traits. Root hair density of this *loss-of-function* mutant was significantly increased in the presence of P_i_ by 35% and by 29% in scarce P_i_, compared to the wild type Col-0 (Fig. 2A). In addition, root hair length increased significantly in the mutant, but was not different between control conditions and local absence of P_i_ (Fig. 2B).

The root hair density and length were also measured in an accession that is strongly stimulated by −P_i_ (Ga-0) and in an accession with reduced root hair length and density under −P_i_ conditions (Kondara). Their hair patterns were significantly different to Col-0 (Figs.2A and 2B). Interestingly, the root diameter was similar in most genotypes, but increased in tetraploid Col-0 (4×), especially in the absence of P_i_ (Fig. 2C) and Ga-0.

### Primary root morphological differences in accessions and *wrky6-3*

**Figure 3:**
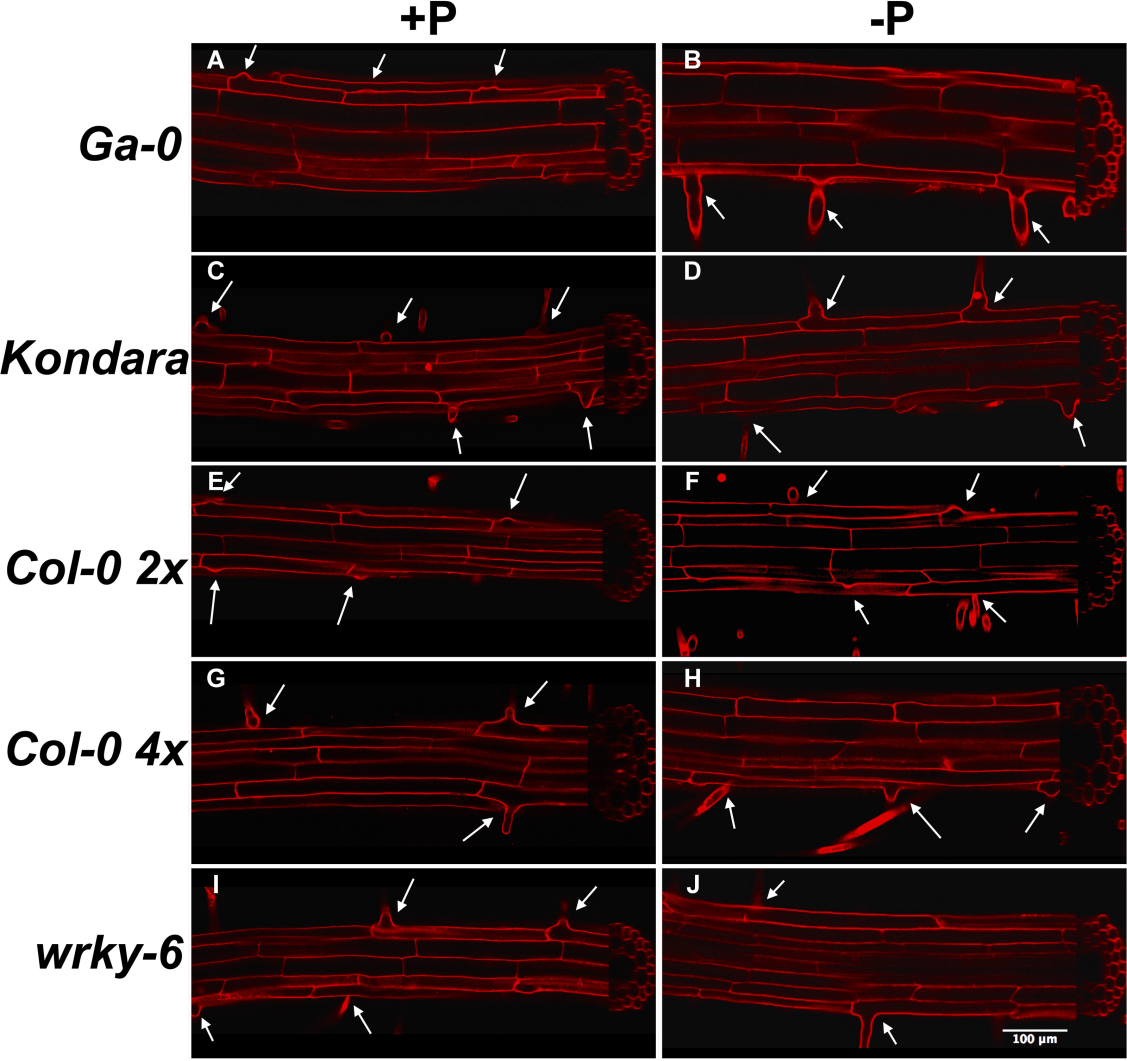
Longitudinal and radial root phenotypes in +P_i_ (A, C, E, G, I) and −P_i_ (B, D, F, H, J) conditions. Propidium iodide stained roots, 1 cm behind the root tip of Ga-0 (A, B), Kondara (C, D), Col-0 2×(E, F), Col-0 4×(G, H), *wrky6-3 (I, J).* Arrows indicate root hairs or root hair bulbs. Scaling bar: 100 µm.

Because different root hair density can have various mechanistic reasons, we microscopically compared root cell length, diameter and radial arrangements in diploid and tetraploid Col-0, Ga-0, Kondara and *wrky6-3*. Longitudinal sections of propidium iodide-stained roots revealed the length of rhizodermal cells, diameter of epidermal and cortical cells, as well as the number of neighboring cells in radial sections (Fig. 3). The data revealed that Kondara, an accession that almost completely lacked root hairs when assessed with low light microscopic resolution under −P_i_, clearly had small bulbs of root hair initiations (Fig. 3). Thus, root hairs were initiated similar as in control conditions, but failed to elongate under −P_i_ (Fig. 3).

**Figure 4:**
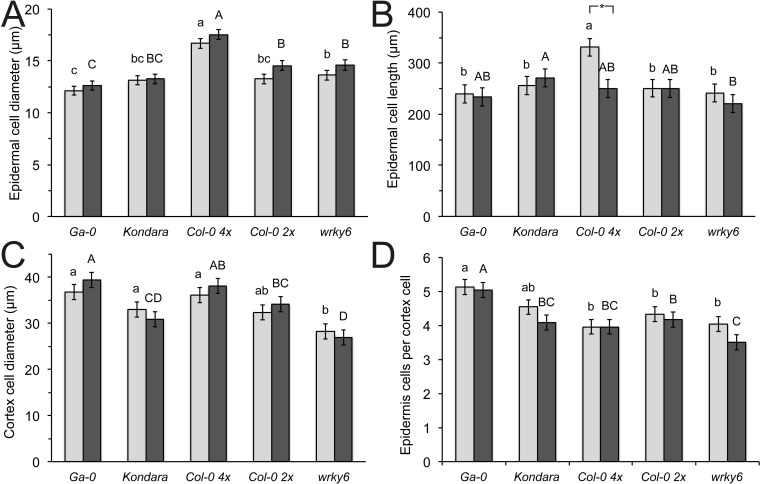
Epidermal and cortical root cell longitudinal length and diameter. (A) Epidermal cell diameter (B) epidermal longitudinal length (C) cortical cell diameter (D) Epidermal cells per cortex cell of Ga-0, Kondara, Col-0 2×, Col-0 4×, *wrky6-3*. Light gray bars: +P_i_, dark grey bars: −P_i_. Error bars indicate standard error. Capital letters indicate significant differences (*p* < 0.05) in +P_i_, lower case letters indicate significant differences (*p* < 0.05) in −P_i_. Significant differences between +P_i_, and −P_i_ are given as *.

Consistent with a thicker root and greater radial root diameter in polyploids, the epidermal cell diameter in Col-0 (4×) was larger than in diploid Col-0 (Fig. 4A). Similarly, the epidermal cells were stretched in Col-0 (4×) control conditions compared to diploids, but were strongly reduced in −P_i_, to similar cell lengths as diploid genotypes. The epidermal length was similar in all diploids, including *wrky6-3* and was not affected by different P_i_ (Fig. 4B). By contrast, the two accessions with the thickest root diameter (Ga-0 and Col-0 4×) also had the largest cortex cell diameters, although these were only significantly different in −P_i_ to Kondara. *wrky6-3* appeared to have significantly smaller cortex cells under −P_i_ than its reference diploid wild type, Col-0. The epidermal cell number per cortex cell did not differ between different P_i_ conditions and was identical between Col-0 2× and Col-0 4×. However, due to the large cortical cells in Ga-0, the ratio between epidermal and cortical cells was significantly increased, while this number was reduced in *wrky6-3* under −P_I_ (Fig. 4D).

## Discussion

### Diversity of root hairs and response to local lack of phosphate

In this study split agar plates were used to determine root hair responses to lack of P_i_. The *Arabidopsis* plants were not systemically P_i_-deficient, as the top part of the root had access to sufficient P_i_. The results are consistent with the idea that different genes control the systemic and local responses to insufficient P_i_ supply (Chiou & Lin, 2011). −P_i_ leads to a correlated increase of root hair density in Col-0 (Bates & Lynch, 2001; Müller & Schmidt, 2004; Narang, Bruene & Altmann, 2000), but not in Kondara. The latter genotype was morphologically not different from Col-0 in cortical or epidermal cells, indicating that other genetic factors, not simply the radial cellular root architecture and morphology, were responsible for genotypic differences. This is corroborated by the fact that root hair bulbs were detected under −P_i_, but hairs did not elongate, leading to macroscopically hairless roots under −P_i_. Ga-0, which had very low hair density under control conditions, but strongly increased the root hair density in −P_i_, had larger cortex cell diameter than Col-0, leading to higher numbers of epidermal cells per cortex cells, both under +*P*_*i*_ and −P_i_. As root hairs are normally only found in the epidermal cells with direct contact to two cortical cells, this can explain the relative low hair cell density in this genotype under control conditions.

Polyploid plants generally have larger cells than diploids and this was confirmed for diploid Col-0 (2×) and the isogenic, colchicine-doubled tetraploid Col-0 (4×). Tetraploid Col-0 had thicker root diameter, which resulted from larger individual cell diameters. The radial ratio of cortical and epidermal cells, however, was unchanged, and the longitudinal epidermal cell length was only increased under +*P*_*i*_. The increased root hair length in tetraploid Col-0 (4×) may thus be related to generally larger root cells, while the unaltered cortex/epidermal cell ratios explain little difference in hair densities.

When the primary root tip comes in contact with a P_i_-deficient medium, primary root growth is reduced, leading to shorter longitudinal elongation of cells (Svistoonoff et al., 2007). A longitudinal reduction in epidermal cells was, however, only found in tetraploid Col-0, but not in the other genotypes, which suggests that the generally small effect of less dense root hairs in the −P_i_ compartment is due to other factors. That polyploidy also caused higher shoot *K*^+^ concentrations and salt resistance in various *A. thaliana* accessions (Chao et al., 2013), may in part be related to root hair-mediated uptake of *K*^+^, another nutrient primarily acquired via root hairs.

### *WRKY6* is associated with root hair traits

Fine association mapping of the root hair density response to low local P revealed associated highly significant SNPs in close proximity to the *WRKY6* transcription factor gene. As SNPs in such a small region are linked, *WRKY6* might be the causal gene associated; causal SNPs are not necessarily closer linked to the trait in GWA or more significant than nearby non-causal SNPs (Atwell et al., 2010). *WRKY6* was initially identified as defense and senescence regulator in the leaves, but is also strongly expressed in the roots (Robatzek & Somssich, 2001; Robatzek & Somssich, 2002). However, *WRKY6* also negatively regulates *PHO1*, a key phosphate transporter gene involved in xylem loading and P_i_ homeostasis, by directly binding to its promoter and inhibiting *PHO1* expression (Chen et al., 2009). This link to P_i_ nutrition is supported by the fact that *WRKY6* is also crucial for arsenate resistance, since arsenate is a structural analog of phosphate and taken up via the same route (Castrillo et al., 2013). P_i_ deficiency releases the *WRKY6* binding to the *PHO1* promoter (Chen et al., 2009) and also directly represses the expression of the phosphate transporter gene *PHT1;1*, a systemically controlled P_i_-starvation induced gene and many other genes (Castrillo et al., 2013). Even further, unrelated functions of *WRKY6* in abscisic acid signaling were recently identified . (Huang et al., 2016), but it is important to note that these phenotypes were identified in ectopic overexpressor mutants, while minor or no phenotypes were identified in *loss-of-function* alleles (Castrillo et al., 2013; Chen et al., 2009; Huang et al., 2016). Although ectopic overexpression, especially of multigene-family transcription factors, is often used to identify novel functions and phenotypes in transgenics, results from such overexpressors may need to be taken with caution, if previously non-expressed genes are activated in cell types in which the relevant gene was not or low expressed in the wild type (Zhang, 2003). Furthermore, the ectopic overexpression *WRKY6*, *WRKY18, WRKY53* and* WRKY70* resulted in similar stunted transgenics, with altered leaf morphology and altered flowering time (Ulker & Somssich, 2004), questioning the specificity of the overexpression approach.

A *loss-of-function* mutant of *WRKY6* had longer and denser root hairs than Col-0, which were also less responsive to the lack of P_i_. *WRKY6* thus appears not only to repress phosphate transporters, but also root hairs. It is strongly up-regulated by a toxic arsenate pulse (Castrillo et al., 2013) and under boron deficiency (Kasajima et al., 2010) further establishing the link to nutrition. Several nutrients thus affect *WRKY6.* Root hairs indiscriminately increase the uptake of several nutrients, thus, *WRKY6* may be generally involved in linking low nutrient availabilities with root hair traits. Small morphological differences to Col-0, especially in the cortex diameter and the reduced epidermal/cortical cell ratio, may partially explain the root hair promotion in *wrky6-3*, as this is expected to lead to more epidermal cells in the H-position. However, *WRKY6* overexpressor mutants clearly had ample root hairs at different P_i_ supply (Chen et al., 2009; Huang et al., 2016). In addition to many other functions, *WRKY6* may act as repressor of root hair density and length, rather than regulator of the P_i_ deficiency response (Huang et al., 2016; Robatzek & Somssich, 2001; Robatzek & Somssich, 2002; Skibbe et al., 2008).

It is noteworthy that the association mapping did not yet identify already known, well-established, crucial genes for root hair traits. Although it is possible that these are not causal for the natural variation seen in* Arabidopsis*, it is likely that larger populations of *Arabidopsis* accessions are mandatory to unravel the genetic architecture of natural variation of root hair traits and the acclimation to low P.

## Conclusions

High-resolution candidate region association mapping, revealed that *WRKY6* is potentially involved in the regulation of root hair traits. A *loss-of-function* mutant had altered radial cellular root morphology, explaining at least part of its higher root hair density and length in −*P*_*i*_.

##  Supplemental Information

10.7717/peerj.2891/supp-1 Data S1 Root hair phenotypes

10.7717/peerj.2891/supp-2 Data S2 Root hair cell raw data

10.7717/peerj.2891/supp-3 Supplemental Information 1 Phenotypes by alleles

## Abbreviations

P: Phosphorus
GWA: Genome wide association
SNP: Single nucleotide polymorphism
MLMM: Multi-locus mixed model
SE: Standard error

## Additional Information and Declarations

### Data Availability

The following information was supplied regarding data availability: The raw data has been supplied as a Supplementary File.
